# Beyond the phonological deficit: Semantics contributes indirectly to decoding efficiency in children with dyslexia

**DOI:** 10.1002/dys.1597

**Published:** 2018-09-21

**Authors:** Robin van Rijthoven, Tijs Kleemans, Eliane Segers, Ludo Verhoeven

**Affiliations:** ^1^ Behavioural Science Institute Radboud University Nijmegen The Netherlands; ^2^ OPM Nijmegen The Netherlands

**Keywords:** dyslexia, semantics, lexical restructuring, pseudoword reading, word reading

## Abstract

The present study explored the direct and indirect contribution of semantic abilities to the levels of phonological and orthographic abilities in children with dyslexia. The semantic abilities of fifty‐five 9‐year‐old Dutch children diagnosed with dyslexia were associated with their phonological abilities (phonological awareness, rapid naming, and verbal working memory) and their word decoding and pseudoword decoding efficiency scores. It was found that children's semantic abilities predicted both word decoding efficiency and pseudoword decoding efficiency indirectly via phonological awareness and rapid naming. These results can be explained in terms of a lexical restructuring account of early reading development; strong semantic abilities provide children with dyslexia with a boost to strengthen their phonological skills and naming skills, which indirectly facilitate their reading development.

## 1 INTRODUCTION

Learning to read is important to become functional literate in today's society. The process of reading involves activation of orthographic, phonological, and semantic features of words (see Coltheart, Rastle, Perry, Langdon, & Ziegler, 2001; Seidenberg & McClelland, 1989). In learning to read, children learn that words constitute of speech sounds that can be represented by letters. By learning the letters and recoding orthographic representations into phonological representations, children become proficient in reading (see Ehri, 2005). Phonological factors are also seen as most critical in predicting early success in reading (Hulme, Snowling, Caravolas, & Carroll, 2005). Research has shown that phonological awareness (PA), rapid automatized naming (RAN; Swanson, Trainin, Necoechea, & Hammill, 2003), and working memory (Jongejan, Verhoeven, & Siegel, 2007) can be seen as important predictors of early word decoding. For dyslexic children, however, the mapping of orthography to phonology is far from trivial. Many of them experience a phonological deficit that obscures the assignment of phonology to orthographic word representations (Snowling & Göbel, 2010). Research also indicated that phonological awareness, rapid naming, and working memory are generally low in children with dyslexia (Tilanus, Segers, & Verhoeven, 2013). Given the presence of a phonological deficit in children with dyslexia, it might well be the case that these children could compensate such deficit with a strongly developed semantic system. It can be assumed that there is a reciprocal connection between semantics and phonology in the mental lexicon (Li, Farkas, & MacWhinney, 2004) and that the development of semantics may give a boost to the development of phonological abilities (Van Goch, McQueen, & Verhoeven, 2014) and thus facilitate the process of learning to read (e.g., Van Bergen et al., 2014). However, the possible beneficial role of semantics in the development of word decoding in children with dyslexia has so far only received scant attention in the literature. Therefore, in the present study, we explored the direct and indirect contribution of semantic abilities to the levels of phonological and orthographic abilities in Dutch children with dyslexia.

## 2 ROLE OF SEMANTICS IN LEARNING TO READ

Learning to read involves grasping the alphabetic principle that involves the acquisition of mappings between orthography and phonology. This requires that children become aware of the sound structure of their language. In the literature, it has indeed been found that phonological abilities predict children's success in learning to read. To begin with, phonological awareness (i.e., the awareness of spoken sounds in language) has been found to be related to the process of mastering the systematic spelling–sound correspondences and to contribute to accurate and fluent word decoding (Melby‐Lervag, Helaas Lyster, & Hulme, 2012). There is also abundant evidence that rapid naming which involves the accurate and efficient storing of detailed phonological or orthographic information is closely related to word decoding (Georgiou, Papadopoulos, Fella, & Parrila, 2012; Norton & Wolf, 2012). And it has also been shown that verbal working memory may constraint the storage of verbal content when excessive demands are being made, such as is the case in word decoding (Swanson, Ashbaker, & Lee, 1996). In the course of reading development, word decoding becomes faster, and gradually, most words are recognized more or less instantly (Bishop & Snowling, 2004; Nation & Snowling, 2004). Becoming a proficient reader requires having high quality lexical representations as claimed by the lexical quality hypothesis (Perfetti, 2007). These representations develop by multiple exposures to words consist of orthographic, phonological, and semantic features (Perfetti & Hart, 2002). For children with dyslexia, however, the acquisition of word decoding is problematic as a consequence of inadequate phonological skills. Already at preliterate age, children at risk for dyslexia have found to be behind in speech decoding (Richardson, Leppänen, Leiwo, & Lyytinen, 2010), phonological awareness, rapid naming, and verbal working memory (Puolakanaho et al., 2007). Their phonological deficit may cause problems in manipulating speech sounds that may hamper the grasping of the alphabetic principle. Moreover, children with dyslexia may stay behind in phonological recoding of written word representations because their phonological lexicon can be considered underspecified (Elbro, 1996; Ramus, 2001).

An important question is how a strong capability in semantics may compensate dyslexic children in reading words and pseudowords. Semantics can, according to the lexical quality hypothesis, be defined as a fuller range of meaning dimensions to discriminate among words in the same semantic field (Perfetti, 2007). Not only vocabulary or the broadness of vocabulary but also the depth of the semantic representations thus may be of importance. The depth of semantic representations is defined as the quality of the meaning network surrounding a word (Nagy & Herman, 1987). Most studies only took the broadness of the semantic lexicon into account and not the depth of the semantic representations (Perfetti & Hart, 2002). Interestingly, the semantic representations of children with dyslexia are quite similar to normal developing children (Nation & Snowling, 1998; Swan & Goswami, 1997), which may provide them with the possibility to use their semantic knowledge as a compensatory mechanism.

Based on the lexical quality hypothesis (Perfetti & Hart, 2002), a direct effect can be expected of semantics on word reading because a better semantic quality of lexical representations may facilitate word identification. And based on the lexical restructuring hypotheses (see Walley, Metsala, & Garlock, 2003), an indirect effect can be expected of semantics on both word and pseudoword reading mediated by phonology. According to this lexical restructuring hypothesis, the development of preliterate phonological abilities in children with dyslexia can be fostered by a strong lexical development. As the numbers of words and their semantic relations increase, a stronger pressure can be hypothesized to make finer phonological distinctions in the mental lexicon. It can thus be assumed that lexical representations start out holistic and become more specified during early and middle childhood (Metsala & Walley, 1998). Indeed, it has been found that children's degree of lexical specificity enhances their phonological awareness (Garlock, Walley, & Metsala, 2001; van Goch et al., 2014) and facilitates their decoding skills (Elbro, Borstrom, & Petersen, 1998). Following a dual route perspective on reading, it can be hypothesized that a more specified lexicon fosters the process of word decoding and visual word recognition. According to the dual route model (see Coltheart et al., 2001), children learn to assign phonology to new words (or pseudowords) by applying the alphabetical principle. In doing so, they store orthographic, phonological, and semantic knowledge in their mental lexicon. Gradually, they become able to address this stored information in the lexical retrieval of frequently encountered words and in a direct route of visual word recognition (Coltheart, 2006). It can thus be expected that semantic knowledge may foster word decoding indirectly via lexical retrieval (i.e., rapid naming) or directly via word recognition.

Neurocognitive research has indeed evidenced that poor readers rely to a greater extent on their semantic lexicon when it comes to word decoding (Shaywitz et al., 2003). Children with dyslexia may thus compensate for their weak orthographic and phonological representations by using their broad and deeply developed semantic knowledge when combining graphemes and phonemes to a recognizable meaningful word. Because the lexical representations of semantic knowledge and phonological knowledge seem to be reciprocally connected, a broad and deep semantic knowledge can be considered useful when phonological representations are less developed (Li et al., 2004). Behavioural evidence for the role of semantics in word decoding has also been found by Nation and Snowling (2004), who showed that weaker semantic skills related to lower word decoding skills, and Ouellette and Beers (2010) who found semantic skills to predict decoding in Grade 6 and irregular word recognition in Grades 1 and 6. There is also evidence that children with dyslexia compensate for their poor decoding skills by using the semantic context during text reading (Nation & Snowling, 1998). Furthermore, a study of Van Bergen et al. (2014) showed verbal IQ (defined by expressive vocabulary, expressive syntax, and comprehension) to be uniquely related to later monosyllabic word decoding in 4‐year‐old children who go on to develop dyslexia. Besides a direct effect of semantic representations on pseudoword and word decoding, also indirect effects have been shown in the literature. Swanson et al. (2003) showed that semantic skills predicted phonological awareness and rapid naming as well as word decoding. In a similar vein, Torppa, Lyytinen, Erksine, Eklund, and Lyytinen (2010) showed that receptive and productive semantic skills in Scandinavian children at familial risk for dyslexia predicted their word decoding through phonological awareness, letter naming, and inflectional morphology.

## 3 THE PRESENT STUDY

To sum up, it has been made clear that orthography, phonology, and semantics are of main importance for learning to read words. Throughout the grades, children develop fully word representations in order to become fluent readers. Although children with dyslexia are in need for more compensation in phonological recoding, it remains unclear for this specific group of readers whether the full semantic lexicon (depth and broadness of the lexicon) could partly compensate for a weak phonological component in building orthographic representations. It is still unclear whether the role of semantics is in assigning phonology to new orthographic representation as tapped with pseudoword reading or in addressing phonology in direct word recognition. An indirect effect of semantics via phonological awareness and rapid naming on word and pseudoword decoding could be predicted in the former case, a direct effect of semantics on word decoding in the latter case. Therefore, in the present study, we investigated the direct and indirect effects of semantics on pseudoword and word decoding within a group of Dutch children with dyslexia, taken into account phonological awareness and rapid naming. In contrast with previous research, in the present study, semantics is defined as much broader than just vocabulary. It is generally known that semantics involves more than vocabulary alone, and especially because the specificity and redundancy of the lexical representations seems to be important (Perfetti & Hart, 2002), a broad operationalization definition of semantics was used, including both lexical knowledge and comprehension skill. Starting from the question to what extent semantics in children with dyslexia would foster their decoding skills, we expected to find pseudoword and word decoding to be both directly and indirectly (via phonological awareness and rapid naming) predicted from semantic knowledge.

## 4 METHOD

### 4.1 Participants

Participants were Dutch children diagnosed with developmental dyslexia who received an in‐service reading and/or spelling intervention in a clinic for assessment and intervention for children with learning difficulties. For the purpose of this study, 99 files of Dutch children were collected from the clinic. Due to missing data and different instruments, a group of 55 children (36 boys and 19 girls) with Dutch as their first language remained for this study. For an anticipated medium effect size (in terms of Cohen's *f* ^2^) of 0.15 for each path including three predictors (semantics, PA, and RAN), a representative group of 55 participants was needed to have sufficient power (>0.80) at the 0.05 level (two tailed) according to calculations as recommended by Faul, Erdfelder, Lang, and Buchner (2007).

All children had been referred to a reading clinic by their parents and teachers. Teachers had to prove resistance to treatment (after 10‐ to 12‐week interventions) and constant weak performances for 1.5 years (word reading scores below 10th percentile or below 15th percentile combined with spelling scores below 10th percentile) for these children. All children were diagnosed with severe dyslexia and received in‐service reading and/or spelling interventions. The mean age of this group of children during assessment was 8.55 years (*SD* = 1.051). Children were in Grade 2 (*n* = 16), Grade 3 (*n* = 25), Grade 4 (*n* = 10), Grade 5 (*n* = 3), and Grade 6 (*n* = 2). Out of the group of 55 children, 15 children attended the same class an extra year. Parents gave active consent to let their child participate in the present research. Because of the large variation in age, age was included as a covariate in the analyses.

### 4.2 Measures

#### 4.2.1 Reading measures

##### Pseudoword decoding

Pseudoword decoding was measured by the “Klepel” (Van den Bos, Lutje Spelberg, Scheepstra, & de Vries, 1994). In this task, the child had to read as many meaningless words correctly as possible within a time limit of 2 min. The card contained 116 unrelated nonwords that have the same structure as meaningful words. Words become more difficult gradually from one syllable (“taaf”) up to five syllables (“nalleroonplinteng”). Efficiency measure (the number of words correctly read within 2 min) was calculated. The reliability of this measure differs per age but is at least 0.89 (Van den Bos et al., 1994).

##### Word decoding

Word decoding was measured by the “Brus One Minute Test” (Brus & Voeten, 1973) In this task, the child had to read as many meaningful words correctly as possible within a time limit of 1 min. The card contained 116 unrelated words. Words become more difficult gradually from one syllable (“waar” [true]) up to four syllables (“tekortkoming” [shortcoming]). Efficiency measure (the number of words correctly read within 1 min) was calculated. The reliability of this measure differs per age but is at least 0.87 (Van den Bos et al., 1994).

#### 4.2.2 Precursor measures

##### Phonological awareness (PA)

PA was measured by adding the z‐scores of two subtests from the “Screening Test for Dyslexia” (Kort, Schittekatte, van den Bos, et al., 2005). First, during “Phoneme Deletion”, the child had to omit a phoneme from an orally presented word and speak out the remaining word (e.g., “da*k*” [roof] minus *k* [f] is “da” [roo]). Testing was terminated after four consecutive mistakes. The reliability differs per age but is at least 0.59 (Kort, Schittekatte, van den Bos, et al., 2005). Second, during the subtest, “Spoonerism” (Kort, Schittekatte, van den Bos, et al., 2005) the child had to switch the first sounds of two words (e.g., “*J*ohn *L*ennon” becomes “*L*ohn *J*ennon”). Testing was terminated after five consecutive mistakes. The reliability differs per age but is at least 0.60 (Kort, Schittekatte, van den Bos, et al., 2005). In both tests, all correctly formed words were counted.

##### Rapid automatized naming (RAN)

RAN was measured by adding the z‐scores of two subtests from “Continuous Naming and Reading Words” (Van den Bos & Lutje Spelberg, 2007). First, during “Naming Letters,” the child had to read out loud 50 letters. Second, during “Naming Digits,” the child had to read out loud 50 digits. The child was asked to name these visual stimuli as fast as possible. The time in seconds, needed to finish each subtest, was used for analysis. A low score therefore represents a good performance on this task. The reliability of this measure differs per age but is at least 0.75 (Van den Bos & Lutje Spelberg, 2007).

##### Verbal working memory

Verbal working memory was measured using the backward task of the Number Recall subtest from the Wechsler Intelligence Scale for Children (WISC‐III^NL^; Kort, Schittekatte, Dekker, et al., 2005). In this task, the experimenter pronounces sequences of digits that the child was asked to repeat in backward order. Testing was terminated after two consecutive mistakes. The number of correctly recalled sequences was counted. The reliability of this measure differs per age but is at least 0.50 (Kort, Schittekatte, Dekker, et al., 2005).

##### Semantics

Semantics were measured by adding the *z*‐scores of four subtests from the WISC‐III^NL^ (Kort, Schittekatte, Dekker, et al., 2005). First, during “Information,” the child has to answer verbally asked questions to test their general knowledge about events, objects, places, and people. Based on the manual, the child received zero, one, or two points for each item. Testing was terminated after five consecutive mistakes. All points were counted afterwards. The reliability differs per age but is at least 0.64 (Kort, Schittekatte, Dekker, et al., 2005). Second, during “Similarities,” the child has to name the similarity between two concepts. Based on the manual, the child received zero, one, or two points for each item. Testing was terminated after four consecutive mistakes. All points were counted afterwards. The reliability differs per age but is at least 0.65 (Kort, Schittekatte, Dekker, et al., 2005). Third, during “Productive vocabulary,” the experimenter pronounces a word and the task of the child was to define the given word. Based on the manual, the child received zero, one, or two points for each item. Testing was terminated after four consecutive mistakes. All points were counted afterwards. The reliability differs per age but is at least 0.77 (Kort, Schittekatte, Dekker, et al., 2005). Fourth, during “Comprehension,” the experimenter asked questions about social situations or common concepts. Based on the manual, the child received zero, one, or two points for each item. Testing was terminated after four consecutive mistakes. All points were counted afterwards. The reliability differs per age but is at least 0.73 (Kort, Schittekatte, Dekker, et al., 2005). Kaufman (1975) already showed that these four measures together form a factor named “verbal comprehension.” To confirm this factor within this specific group a principal component analysis with varimax rotation on all subtests of WISC‐III^NL^ is added. This showed a four‐factor distinction, together explaining 75.79% of variance in intelligence measures. The first factor included semantics (information 0.810, similarities 0.807, vocabulary 0.874, and comprehension 0.839), the second factor visual spatial abilities (block design 0.847, visual puzzles 0.772, incomplete drawings 0.583, and pictures organization 0.492), the third factor working memory (digit span forwards 0.648 and digit span backwards 0.745), and the fourth factor processing speed (substitution 0.914 and symbol search 0.931). To make the impact of all variables equal in this research, factor scores were not included in the analyses.

##### Perceptual organization

Perceptual organization was measured by adding the *z*‐scores of four subtests from the WISC‐III^NL^ (Kort, Schittekatte, Dekker, et al., 2005). First, during “Incomplete drawings,” the child has to name or point at a missing part in a drawing of familiar objects or situations within 30 s. Testing was terminated after five consecutive mistakes. The child received one point for each item. All points were counted afterwards. The reliability differs per age but is at least 0.54 (Kort, Schittekatte, Dekker, et al., 2005). Second, during “Picture organization,” the child has to put pictures in the right order to make the story depict right. The child was asked to do this as quickly as possible. Testing was terminated after three consecutive mistakes. The first two items are scored two points at first attempt and one point at second attempt. The other items were given zero, two, three, and four of five points based on accuracy and the time the child needs to order the pictures. All points were counted afterwards. The reliability differs per age but is at least 0.65 (Kort, Schittekatte, Dekker, et al., 2005). Third, during “Block design,” the child had to reconstruct patters of two, four, and later nine blocks shown on a picture. The child was asked to do this as quickly as possible. Testing was terminated after two consecutive attempts. The first three items are scored two points at first attempt and one point at second attempt. The other items were give four, five, six, or seven points based on accuracy and the time the child needs to reconstruct the pattern. All points were counted afterwards. The reliability differs per age but is at least 0.71 (Kort, Schittekatte, Dekker, et al., 2005). Fourth, during “Visual puzzles,” the child has to make five puzzles of everyday objects. Testing was terminated after four consecutive mistakes. Each right connection between puzzle pieces was given one point. Extra points were given when less time was needed to make the puzzle with a maximum of 10 points. All points were counted afterwards. The reliability differs per age but is at least 0.40 (Kort, Schittekatte, Dekker, et al., 2005).

### 4.3 Procedure

The current study was based on existing data collected by a clinic for assessment and intervention of children with learning disorders. Between 2009 and 2013, data were filed in this clinic. The following procedures were followed: Assessment started with parents and teacher filling in questionnaires about current problems and child's development. Afterwards, parents were invited for an interview at the clinic. Both the questionnaires and the interview were in order to rule out other explanations for reading and spelling problems. Assessment took place at a clinic for assessment and intervention of children with learning disorders. Parents brought their children to the clinic in Nijmegen (the Netherlands), and MSc‐graduated clinicians tested the children individually in quiet rooms. Mostly the assessment took two consecutive mornings from about 9.00 a.m. until 12.00 a.m. including breaks. After assessment, clinical reports were written by the MSc‐graduated clinicians. The continuity of quality was guaranteed by supervision of certified clinical health psychologists. The stored files were used for this study.

## 5 RESULTS

Table 1 presents the descriptive statistics of all standardized measures. Both raw scores and age‐related scaled scores are given.

**Table 1:** Descriptive statistics for all participants including raw scores and age‐based scaled scores

	*n*	M	*SD*	Min.	Max.
PA					
Spoonerism					
Raw scores	55	2.93	3.293	0	11
Scaled scores	55	7.75	2.205	4	13
Phoneme deletion					
Raw scores	55	8.07	2.395	2	12
Scaled scores	55	7.95	2.384	4	15
RAN					
Letter naming					
Raw scores	55	40.58	10.838	24	75
Scaled scores	55	5.53	2.581	1	13
Digit naming					
Raw scores	55	36.64	11.069	22	86
Scaled scores	55	6.45	2.980	1	12
Working memory					
Raw scores	55	4.04	1.360	2	8
Semantics					
Information					
Raw Scores	55	13.00	3.657	7	23
Scaled scores	55	10.67	2.381	6	17
Similarities					
Raw scores	55	14.60	4.875	5	27
Scaled scores	55	12.36	2.895	6	19
Vocabulary					
Raw scores	55	31.05	6.718	19	52
Scaled scores	55	11.55	2.418	8	19
Comprehension					
Raw scores	55	20.96	5.686	5	38
Scaled scores	55	11.67	2.465	4	19
Pseudoword decoding					
Raw scores	55	19.89	9.908	6	50
Scaled scores	55	5.22	1.863	1	8
Word decoding					
Raw scores	55	30.29	13.024	7	62
Scaled scores	55	4.51	1.804	1	8

*Note*. All scaled score population means are 10 and standard deviations are 3. Working memory is only described in terms of means. PA: phonological awareness; RAN: rapid automatized naming.

Prior to the analyses, all scores (except for age) were standardized using *z*‐scores. *Z*‐scores were calculated by subtracting the mean raw score from each value and then divided that by the standard deviation. A composite score of these *z*‐scores was computed for PA, RAN, semantics, and perceptual organization. RAN is the only variable in which a negative score means a better performance because this is the only variable were time is included. In Table 2, Pearson correlations are given. All predictor measures were significantly related to word decoding and pseudoword decoding. Furthermore, semantics was found to be significantly related to PA and RAN. Of the covariates, age and perceptual organization were significantly related to word decoding and pseudoword decoding.

**Table 2:** Pearson correlations between the predictor measures (i.e., performal IQ scores, age, working memory, PA, RAN, and semantics) and criterion measures (i.e., pseudoword decoding and word decoding; *n* = 55)

	1	2	3	4	5	6	7	8	9	10	11	12
1. Perceptual organization	‐											
2. Age	0.504**	‐										
3. Working memory	0.131	0.229	‐									
4. PA	0.297*	0.391**	0.175	‐								
5. Spoonerism	0.330*	0.515**	0.265	0.875***	‐							
6. Phoneme deletion	0.176	0.146	0.028	0.850***	0.489***	‐						
7. RAN	−0.219	−0.446***	0.020	−0.204	−0.160	−0.194	‐					
8. Letter naming	−0.262	−0.494***	−0.099	−0.199	−0.189	−0.153	0.930***	‐				
9. Digit naming	−0.146	−0.367**	0.135	−0.181	−0.109	−0.207	0.932***	0.733***	‐			
10. Semantics	0.622**	0.679***	0.141	0.357**	0.462***	0.142	−0.365**	−0.387**	−0.294*	‐		
11. Pseudoword decoding	0.475**	0.645***	0.297*	0.510***	0.556***	0.326*	−0.480***	−0.535***	−0.497***	0.549***	‐	
12. Word decoding	0.550**	0.807***	0.234	0.516***	0.483***	0.394*	−0.544***	−0.485**	−0.408**	0.667***	0.841***	‐

*Note*. PA: phonological awareness; RAN: rapid automatized naming.

* *p* < 0.05.

** *p* < 0.01.

*** *p* < 0.001.

To answer the research questions, two regression based mediation analyses were conducted with the process add‐on in SPSS (Hayes, 2013). Bootstrapping was set at 5,000 cycles, as recommended by Hayes (2013). In mediation models, the addition of the direct effect (c′) and the indirect effect (ab) form the total effect (c) of a certain independent variable on a dependent variable. The indirect effect (ab) is formed by multiplying the effect of the independent variable on the mediator (a) and the effect of the mediator on the dependent variable (b). A total effect may not reach significance, even when the direct effect is significant (Hayes, 2009), which could be cue to a relatively small sample size (Shrout & Bolger, 2002). The final model of this paper includes two statistical models with different dependent variables. In the first model, word decoding was the dependent variable, and in the second model, pseudoword decoding was the dependent variable. In both analyses, verbal working memory, perceptual organization, and age were added as covariates. To ensure that the analyses had enough statistical power, the covariates were added in the models of the dependent variable only. Therefore, the direct and indirect effect do not add up to the total effect.

The results of the first mediation analysis with word decoding as the dependent variable showed no significant total or direct effect of semantics (c = 0.0255, *SE* = 0.0248, 95% CI [−0.0316, 0.0825]; *p =* 0.374, c′ = 0.0414, *SE* = 0.0319, 95% CI [−0.0226, 0.1055]; *p* = 0.200). Semantics, however, was significantly related to PA and RAN. Indirect effects of semantics were found via PA (ab = 0.1712, *SE* = 0.0615, 95% CI [0.0478, 0.2945]) and RAN (ab = −0.1879, *SE* = 0.0657, 95% CI [−0.3197, 0.0560]). The total indirect effect of semantics was significant (0.0421, *SE* = 0.0173, 95% CI [0.0162, 0.0856]). The relation between semantics and word decoding was partially mediated by PA and RAN. The total effect of the model is *R* ^2^ = 0.691 (MSE = 0.3052, *p* < 0.001). Age had a significant total effect on word reading (0.0485, *SE* = 0.0086, 95% CI [0.0313, 0.0657]). There were no significant effects of covariates perceptual organization and verbal working memory.

**Figure 1:**
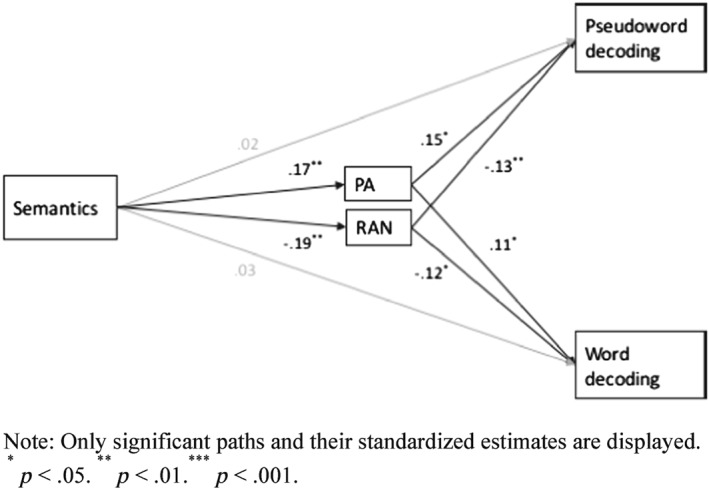
Path diagram for the predictive role of the semantic knowledge on pseudoword, word decoding and the mediators PA and RAN

The results of the second mediation analysis with pseudoword decoding as the dependent variable showed no significant total or direct effect of semantics (c = 0.0371, *SE* = 0.0423, 95% CI [−0.0478, 0.1220]; *p =* 0.384, c′ = 0.0183, *SE* = 0.0389, 95% CI [−0.0599, 0.0964]; *p =* 0.641). Semantics, however, was significantly related to PA and RAN. Indirect effects were found via PA (ab = 0.0248, *SE* = 0.0165, 95% CI [0.0017, 0.0697]) and RAN (ab = 0.0250, *SE* = 0.0114, 95% CI [0.0079, 0.0542]). The total indirect effect of semantics was significant (0.0498, *SE* = 0.0210, 95% CI [0.0172, 0.0995]). The relation between semantics and word decoding was partially mediated by PA and RAN. The total effect of the model is *R* ^2^ = 0.476 (MSE = 0.5351, *p* < 0.001). Age did have a significant total effect on pseudoword decoding (0.0351, *SE* = 0.0113, 95% CI [0.0123, 0.0578]). There were no significant effects of covariates perceptual organization or verbal working memory. Figure 1 depicts the final models.

## 6 CONCLUSIONS AND DISCUSSION

The main goal of the present study was to investigate the direct and indirect effect of semantic knowledge on pseudoword and word decoding within a group of Dutch children with dyslexia. It was questioned how semantic knowledge in children with dyslexia predicts pseudoword and word decoding directly and indirectly.

To begin with, significant indirect effects of semantic knowledge were found on both pseudoword decoding efficiency and word decoding efficiency. In line with findings of Torppa et al. (2010), we found that children's semantic knowledge has a significant effect on both phonological awareness and rapid naming. Just as found by Metsala (1999), we found that a more specific and redundant lexicon is related to phonological awareness. This indicates that the lexicon could facilitate phonological awareness. Although the relation between semantic knowledge and phonological awareness is generally assumed to be reciprocal (Castles & Coltheart, 2004; Perfetti, Beck, Bell, & Hughes, 1987), in the early stages of reading, semantic processing influences reading development and not vice versa (see Verhoeven, van Leeuwe, & Vermeer, 2011). Children with dyslexia are still in their early stages of reading, and thus, these results fit prior research. We also found an effect of semantic knowledge on rapid naming as an index of efficiency in lexical retrieval. Importantly, besides direct effects of semantics on phonological awareness and rapid naming, we also found a significant effect of the latter two abilities on both pseudoword decoding efficiency and word decoding efficiency. In other words, we found a relation between semantic knowledge and word and pseudoword decoding efficiency via phonological awareness and rapid naming. The found indirect effect of semantics via rapid naming is in line with Wolf, Bally, and Morris (2016).

However, we found no significant total or direct effect of semantic knowledge on word decoding and pseudoword decoding. This was not expected based on the triangular reading model claiming that orthographic representations are linked to both phonological and semantic representations (Seidenberg & McClelland, 1989). Especially for children with dyslexia, who experience weak orthographic‐phonological connections (Wimmer & Schurz, 2010), the use of semantic knowledge could be considered commendable. The finding that there was no impact of broad and deep semantic knowledge on word decoding for children with dyslexia is not in line with previous research (i.e., Van Bergen et al., 2014). The effect of semantic knowledge on word decoding is also not commensurate with the lexical quality hypothesis claiming that proficient reading requires redundant lexicalized word representations (Perfetti & Hart, 2002). It can tentatively be explained from the fact that the children with dyslexia are only in the beginning of their reading development with a primary focus on phonological recoding which is heavily influenced by phonological awareness and rapid naming, explaining the indirect effect we found of these abilities on children's decoding efficiency.

Of course, the present study can only be seen as a first step in uncovering the role of semantics in dyslexic children's processes of learning to read. It should be acknowledged that the present data were cross‐sectional and that causal conclusions as regards relations between semantics and decoding efficiency measures cannot be drawn. It would be informative to know whether normal reading peers to a certain extent show an effect of semantic knowledge as well. Future studies following a longitudinal design comparing dyslexic and typical readers are needed to arrive at final answers as regards the role of lexical semantics in learning to read. A second point to mention is the large variability of age in the sample. Therefore, age is included as a covariate on the dependent variable. One could argue that age should be included as a covariate on the independent variables as well. This would lead to underpowered analyses. Therefore, age is not included on all variables; hence, results should be interpreted with caution. More research with more power is necessary to disentangle the effect of age on the model. Furthermore, it should be considered to use measures of receptive and not expressive semantics instead of measures that require word retrieval and verbal formulation because dyslexia and specific language impairment (SLI) are comorbid developmental language disorders (Catts, Adlof, Hogan, & Weismer, 2005). Besides semantic knowledge also the role of retrieval could be taken into account. A final point to make is that although the results of the factor analysis indicated that the four subtests that measured lexical semantics loaded on one and the same factor, it could be argued that the subtests similarities and productive vocabulary are conceptually more related to semantics as compared with the other two subtests (see Burton et al., 2001; Cohen, 1959). We therefore conducted a secondary analysis with only these two measures (i.e., similarities and productive vocabulary). The overall conclusions remain the same: Semantics were still indirectly related to word decoding and pseudoword reading. The present study leads to implications for future research. It is shown that semantics are indirectly related to word and pseudoword reading, via their relation to phonological awareness and rapid naming. This association could indicate that semantics are a facilitating factor for forming fine‐grained phonological and orthographic representations and so indirectly influence reading development. A recent study by Van Gorp, Segers, and Verhoeven (2016) has indeed shown that semantic categorization in addition to feedback and motivation in a word decoding training task may yield important effects on decoding efficiency in poor readers in second grade. To find out to what extent the effects are due to semantic categorization, feedback or motivational aspects of the training are object to future research.

Overall, the present findings indicate that the semantic lexicon of children with dyslexia could contribute to pseudoword and word decoding efficiency indirectly. The indirect effects point to the fact that lexical specificity may help dyslexics to become better phonologically aware (see Metsala, 1999) and to become better in lexical retrieval (see Wolf et al., 2016), both of which have a positive impact on decoding efficiency. Furthermore, it fits the lexical restructuring hypothesis in which lexical semantic subsystems could foster the phonological abilities and thereby word and pseudoword reading. Regarding the dual route model, it is possible that the better specified lexicon is the product of the nonlexical route but also partly by semantic development. The finding that there are no direct effects of semantics on decoding measures point to the fact that children with dyslexia do not directly profit from a strong semantic knowledge component in learning to read.

Based on these results, it seems possible that dyslectic children compensate their weak ability to form phonological and orthographic representations by use of their semantic abilities as reasoned in the lexical quality hypothesis and lexical restructuring hypothesis. Even though the effect of semantics was small and indirect, these findings show the relevance of a broad and deep semantic knowledge in the reading development of children with dyslexia. Semantics seems to be a variable that should be taken into account in further research regarding reading.
